# Interpreting antiviral effects: from experimental measurements to mechanistic viral dynamics

**DOI:** 10.3389/fmolb.2026.1995001

**Published:** 2026-10-13

**Authors:** Ashot Nersisyan

**Affiliations:** Faculty of Biology and Biotechnology, HSE University, Moscow, Russia

**Keywords:** antiviral activity, antiviral drug development, *In vitro* models, mathematical modeling, parameter identifiability, pharmacodynamics, viral dynamics, viral load

## Abstract

Antiviral activity is commonly summarized using endpoint measures such as EC50 or EC90, yet these quantities emerge from a dynamic biological system and depend on viral replication kinetics, host-cell properties, experimental design, and the assay used to quantify infection. Infectious-virus titers, viral RNA, infected-cell fractions, viability measurements, and immune readouts capture different components of the infection process and are therefore not interchangeable measures of “viral load.” Mechanistic viral-dynamics models provide a framework for linking such observations to underlying processes, including cell infection, intracellular replication, virus production, cell loss, and host-mediated control. However, the biological information that can be recovered from these models is fundamentally constrained by experimental observability and parameter identifiability. This limitation becomes increasingly important across biological scales: *in vitro* systems permit dense multimodal measurements and controlled perturbations, whereas animal and human studies provide greater physiological relevance but substantially sparser and more indirect observations. Here, we review how experimental readouts map onto viral-dynamics models, how model structure and parameter inference affect mechanistic interpretation, and how controlled *in vitro* systems can be used to distinguish competing mechanisms of infection and antiviral action. We further discuss examples in which viral-dynamics modeling has contributed to antiviral pharmacology and regimen selection. Together, these considerations support a view of antiviral effects as context-dependent dynamic phenotypes that require joint interpretation of measurements, models, and experimental design.

## Introduction

Antiviral potency is conventionally quantified using the half-maximal effective or inhibitory concentration (EC50 or IC50), while EC90 and related measures are frequently used when a higher level of viral inhibition is of interest. These parameters provide a convenient way to compare candidate compounds and are routinely used during antiviral screening and lead optimization. However, EC50 is sometimes implicitly considered an intrinsic characteristic of a drug-virus pair. This interpretation is problematic for antiviral agents because virus replication is a dynamic process. In contrast to many biochemical assays, cell-based antiviral assays typically measure the cumulative outcome of multiple processes, including cell infection, intracellular virus replication, production of progeny virions and subsequent rounds of infection. Consequently, the measured antiviral effect depends not only on the molecular interaction between the drug and its target, but also on the dynamics of the experimental system. This problem was highlighted more than 2 decades ago by Ferguson et al., who demonstrated that conventional endpoint assays provide cumulative measures of antiviral inhibition that depend on assay design and may therefore have limited correspondence to instantaneous antiviral activity (Ferguson et al., 2001).

One immediate consequence is that measured antiviral potency depends on the time at which the experiment is evaluated. A compound that partially decreases the rate of viral replication may produce only a moderate difference from the untreated control during the first replication cycle, whereas the same effect can result in a substantial difference after several subsequent cycles. Thus, EC50 estimated at 24 h post-infection does not necessarily represent the same pharmacodynamic quantity as EC50 estimated at 48 or 72 h. This effect was recently quantified by Wang et al. using a mechanistic model of an *in vitro* antiviral assay. Variation in sampling time resulted in more than a 15-fold difference in the estimated EC50 over the conditions considered in their simulations (Wang et al., 2026). The problem is particularly relevant for multi-cycle assays, where relatively small differences in replication at each cycle can accumulate over time.

Importantly, this dependence is not determined by experimental timing alone. The underlying kinetics of the virus-cell system also affect the apparent potency of an antiviral compound. Parameters such as the rate of cell infection, duration of the eclipse phase and rate of virus production determine how rapidly infection propagates through the culture and, consequently, how strongly a given molecular inhibition is amplified at the population level. Wang et al. demonstrated that variation in infection and virus production rates produced more than 20-fold and 50-fold changes in apparent EC50, respectively, while simultaneous changes in these parameters resulted in differences approaching three orders of magnitude (Wang et al., 2026). Similar considerations have previously been demonstrated for HIV, where experimentally measured IC50 values were shown to depend not only on the intrinsic properties of the antiviral compound but also on the kinetics of the viral life cycle (Sedaghat and Wilke, 2011). Thus, differences in EC50 between viral strains or variants do not necessarily indicate proportional differences in their molecular susceptibility to a drug.

The experimental context provides an additional source of variability. Multiplicity of infection, initial cell number, cell density, timing of treatment, duration of drug exposure and the number of viral replication cycles can all influence the observed antiviral effect. For example, the activity of influenza neuraminidase inhibitors was shown to decrease with increasing multiplicity of infection and to depend strongly on the time of drug administration relative to infection (Smee et al., 2001). Cell type can be equally important, particularly for compounds that require intracellular activation. Remdesivir provides a useful example. Comparison of remdesivir and its parent nucleoside GS-441524 across Vero, Calu-3 and Caco-2 cells demonstrated substantial cell-type-dependent differences in antiviral activity that were associated with differences in intracellular formation of the active nucleoside triphosphate (Tao et al., 2021). Therefore, an EC50 measured in a particular cell line characterizes not only the drug and virus but also the cellular system in which the drug is metabolized and the virus replicates.

Furthermore, the EC50 alone does not fully characterize a concentration-response relationship. Two compounds with similar EC50 values may produce substantially different levels of inhibition at concentrations above the EC50 if their dose-response curves have different slopes. This was demonstrated particularly clearly for antiretroviral drugs. Shen et al. showed that the slope of the dose-response curve differed systematically among HIV drug classes and strongly affected the antiviral inhibition achieved at clinically relevant concentrations (Shen et al., 2008). The authors therefore proposed instantaneous inhibitory potential as a measure that incorporates both drug concentration and dose-response slope. These results further illustrate that reduction of antiviral activity to a single concentration threshold can obscure important pharmacodynamic differences between compounds.

The interpretation becomes still more complicated when antiviral potency is translated between biological scales. An EC50 obtained in cell culture should be considered a property of a particular drug-virus-cell-assay system rather than a direct estimate of potency in an infected organism. Transition from cell culture to an animal model introduces systemic pharmacokinetics, protein binding, tissue distribution, heterogeneous target-cell populations and host immune responses. Translation to humans introduces additional uncertainty because both drug exposure and viral replication may differ substantially among anatomical compartments. Moreover, for antivirals acting on intracellular targets, plasma drug concentrations may not accurately represent pharmacologically active intracellular exposure. This is particularly evident for nucleoside analogues, which require intracellular conversion to active metabolites; for antiretroviral nucleoside reverse-transcriptase inhibitors, intracellular metabolite concentrations have in some studies correlated with virological efficacy more strongly than plasma concentrations (Bazzoli et al., 2010). Similar pharmacokinetic and pharmacodynamic considerations have been emphasized in the evaluation of antivirals against SARS-CoV-2, where uncertainty in drug disposition and effective exposure at relevant sites of infection complicates direct translation of *in vitro* activity to clinical efficacy (Zeitlinger et al., 2020).

For these reasons, a change in viral load measured in an infected individual should not generally be interpreted as a direct measurement of intrinsic antiviral potency. The observed response to treatment emerges from the interaction between drug-mediated inhibition and the dynamics of the infected system. Classical studies of HIV demonstrated that the decline in plasma virus following antiviral treatment reflects rapid virion clearance and loss of productively infected cells, allowing these otherwise unobserved processes to be quantified from treatment-induced dynamics (Perelson et al., 1996). Subsequent work in HIV and HCV further demonstrated how mechanistic models can separate antiviral drug efficacy from viral and infected-cell kinetic parameters (Perelson and Ribeiro, 2008). Thus, even when treatment produces a readily measurable change in viral load, the magnitude and shape of that change cannot be attributed to a drug-specific parameter alone.

We therefore propose that experimentally observed antiviral potency should be considered a dynamic phenotype emerging from the interaction between drug action, viral replication kinetics, host-cell biology, and the design and timing of the experiment. This view does not diminish the utility of conventional EC50 or EC90 measurements, which remain indispensable for antiviral screening and comparison of candidate compounds. Rather, it defines the context in which these measurements can be interpreted and highlights the limitations of treating them as context-independent characteristics. This interpretation is consistent with theoretical and experimental work showing that measured antiviral potency depends on both the kinetics of viral replication and the structure of the assay used to quantify inhibition (Ferguson et al., 2001; Sedaghat and Wilke, 2011).

Rather than providing a comprehensive catalogue of viral-dynamics model structures, this review focuses on how experimental measurements determine the mechanistic information that can be recovered from such models. We focus particularly on how this relationship changes across *in vitro*, animal and human systems and on its implications for interpreting antiviral effects.

A further difficulty follows directly from this interpretation. The measured antiviral effect depends not only on the dynamics of the system but also on the experimental quantity used to characterize infection. Infectious-virus titer, viral RNA, reporter activity, infected-cell fraction and cell viability capture different components of the viral life cycle and may respond differently to the same antiviral perturbation. For example, RT-PCR positivity does not necessarily correspond to the presence of cultivable infectious virus (Bullard et al., 2020). The biological meaning of an experimentally derived EC50 therefore depends on the readout used to define antiviral activity, motivating a closer consideration of what antiviral assays actually measure.

## What does an antiviral assay actually measure?

A central difficulty in the interpretation of antiviral experiments is the distinction between the biological state of an infected system and the quantities that are experimentally observed. Even a simple viral-dynamics model may contain populations of uninfected cells, infected cells and free virus, whereas more detailed models can additionally describe intracellular viral RNA, viral proteins, different stages of infected cells, antiviral cytokines or immune-cell populations. Experimental assays, however, generally do not measure these model variables directly. Instead, each assay provides an observable that is related to one or several underlying biological states. Consequently, the frequently used term “viral load” is intrinsically ambiguous: depending on the experimental setting, it may refer to infectious-virus titer, viral genome concentration, antigen abundance or another proxy for infection. The distinction becomes particularly important when experimental measurements are used to infer mechanisms of antiviral action.

*In vitro* infection systems provide the greatest flexibility for measuring different components of viral replication. Infectious-virus production can be quantified using plaque assays, focus-forming assays or endpoint dilution methods such as the 50% tissue culture infectious dose (TCID50). Plaque assays quantify replication-competent virus capable of initiating infection and forming plaques under the particular assay conditions, while TCID50 estimates the dilution at which infection is detected in 50% of inoculated cultures (Bullen et al., 2022). These quantities therefore characterize infectivity rather than the total number of physical viral particles. Moreover, they depend on the susceptible indicator cell line, incubation conditions and criteria used to detect infection. Thus, even an infectious-virus titer should be interpreted as an assay-defined measure of infectious activity rather than a direct count of free virions.

Nucleic-acid-based assays measure a fundamentally different quantity. RT-qPCR and related approaches quantify viral genome copies or specific viral transcripts irrespective of whether these molecules are contained within replication-competent virions. The biological interpretation further depends on the sampled compartment. Extracellular viral RNA can originate from infectious virions, noninfectious or defective particles, and potentially free or cell-associated material released during infection. Intracellular viral RNA instead provides information about events occurring within infected cells and, depending on the virus and target sequence, may reflect incoming genomes, replication intermediates or newly synthesized viral RNA. Accordingly, intracellular and extracellular measurements cannot generally be treated as interchangeable observations of the same model variable. Detailed influenza models, for example, have used measurements of distinct intracellular viral RNA species to constrain transcription and genome-replication processes separately from extracellular virus production (Heldt et al., 2013).

Cell-based measurements provide another complementary view of infection. Viral antigen staining, reporter viruses, microscopy and flow cytometry can quantify the fraction or number of infected cells and therefore provide information more directly related to the infected-cell population. In contrast, viability assays measure the remaining viable cell population and cannot normally distinguish viable infected from viable uninfected cells. Measurements of apoptosis or cytopathic effect similarly characterize consequences of infection rather than viral replication itself. Finally, cytokine concentrations, interferon responses, viral or host protein abundance and transcriptomic measurements can provide observables related to host responses that remain completely hidden when only viral titers are measured. The value of combining such measurements is illustrated by studies in which infectious and total viral loads were measured simultaneously with infected and uninfected cell populations, allowing properties of the infection process that could not be resolved from a single viral measurement to be estimated (Iwami et al., 2012).

The distinction between these modalities is not merely technical, because they can display different temporal behavior. In hospitalized patients with influenza A, for example, infectious-virus titers measured by TCID50 declined faster than viral RNA measured by RT-qPCR, resulting in a time-dependent change in the ratio between RNA copies and infectious virus (Van Wesenbeeck et al., 2015). Comparisons of RT-qPCR and plaque assays for respiratory syncytial virus have likewise shown that gene-copy number and infectious titer are related but represent experimentally distinct quantities (Yamamoto et al., 2018). A mechanistic analysis of SARS-CoV-2 infection and transmission in hamsters similarly found that the relationship between viral RNA and infectious virus changed over time, with high viral RNA levels late in infection providing a poor indicator of transmission (Heitzman-Breen and Ciupe, 2022). This distinction can be particularly important in antiviral studies. A treatment that interferes with virion assembly or maturation may strongly reduce the production of infectious progeny without causing a proportional decrease in viral RNA, whereas a polymerase inhibitor may directly alter intracellular genome synthesis. The apparent magnitude and even the kinetics of an antiviral effect therefore depend on which component of the infection process is measured.

The range of accessible measurements changes substantially when moving from cell culture to an infected organism. Animal models introduce physiological processes absent from conventional cell culture, including systemic pharmacokinetics, tissue structure and a functional immune system, while still permitting experimental control over the time, route and dose of infection. Longitudinal measurements may include nasal or oral viral shedding, blood biomarkers, body weight and clinical scores. At predetermined time points, animals can additionally be sacrificed to quantify viral RNA and infectious virus in multiple organs, perform histopathology and measure local immune responses. SARS-CoV-2 infection in Syrian hamsters provides an illustrative example: studies have simultaneously characterized infectious virus, viral RNA, viral antigen, lung pathology and inflammatory responses at multiple stages of infection (Yang et al., 2021; Mulka et al., 2022).

However, the additional biological realism of animal models comes at the cost of reduced observability. Many of the most informative measurements, including tissue-specific viral titers, histology and local immune responses, require terminal sampling. The resulting time course is therefore frequently reconstructed from different animals sacrificed at different time points rather than from repeated observations of the same infection. Furthermore, the experimental design itself can alter the distribution of infection. In rhesus macaques, for example, intranasal and aerosol inoculation of SARS-CoV-2 produced different patterns of upper- and lower-respiratory-tract replication (Fischer et al., 2024). The sampled anatomical site can also affect the viral dynamics inferred from the same host. Modeling of nasal and throat viral-load measurements from young and aged cynomolgus macaques identified substantial age-associated differences in estimated viral-kinetic parameters from nasal measurements, whereas fewer differences were inferred from throat measurements (Rodriguez and Dobrovolny, 2021). Thus, measurements obtained from an animal model reflect not only the virus and host species but also the experimental and anatomical context in which infection is observed.

The problem becomes still more pronounced in humans. Although human measurements have the highest direct clinical relevance, only a small fraction of the underlying infection process is normally accessible. For respiratory viral infections, longitudinal measurements frequently consist of nasal or nasopharyngeal swabs analyzed by RT-PCR, sometimes complemented by antigen testing or viral culture. Blood samples can provide systemic immune measurements, but susceptible-cell abundance, infected-cell populations, intracellular viral replication and local tissue immune responses are rarely measured repeatedly during an acute infection. Moreover, a respiratory swab samples one accessible anatomical compartment rather than the total amount of virus in an infected individual.

SARS-CoV-2 infection provides particularly clear examples of this limitation. Paired longitudinal sampling has demonstrated that viral RNA kinetics can differ substantially between saliva and nasal swabs, with viral genome loads in saliva frequently rising and peaking earlier than those in nasal samples (Ke et al., 2022; Savela et al., 2022). These observations indicate that even two easily accessible respiratory samples can represent distinct local infection dynamics. Consequently, a nasal viral-load trajectory should not automatically be interpreted as a direct proxy for viral replication throughout the respiratory tract.

The distinction between measured viral RNA and infectious virus is equally important. Prospective daily sampling of SARS-CoV-2-infected individuals has shown that RNA and cultivable infectious virus follow related but nonidentical trajectories (Hakki et al., 2022). In another longitudinal study, the median time from symptom onset to the first negative viral-culture result was approximately 11 days, whereas viral RNA remained detectable for more than 19 days (Drain et al., 2023). Earlier studies similarly demonstrated that RT-PCR-positive respiratory samples do not invariably contain cultivable virus and that the probability of recovering infectious SARS-CoV-2 falls substantially as infection progresses and PCR cycle-threshold values increase (Bullard et al., 2020; Basile et al., 2021). Thus, persistent RNA detection does not necessarily indicate persistent production of replication-competent virus.

Taken together, these experimental settings form an important observability gradient. Cell-culture systems provide comparatively simple biology but allow dense sampling of multiple viral and cellular quantities under tightly controlled conditions. Animal models introduce tissue structure, pharmacokinetics and immunity, but many mechanistically informative measurements become destructive or sparse. Human infection contains the full biological complexity relevant to treatment while providing access to only a limited set of surrogate and compartment-specific measurements. Therefore, experimental observability generally decreases as biological and clinical relevance increases (Figure 1).

**FIGURE 1:**
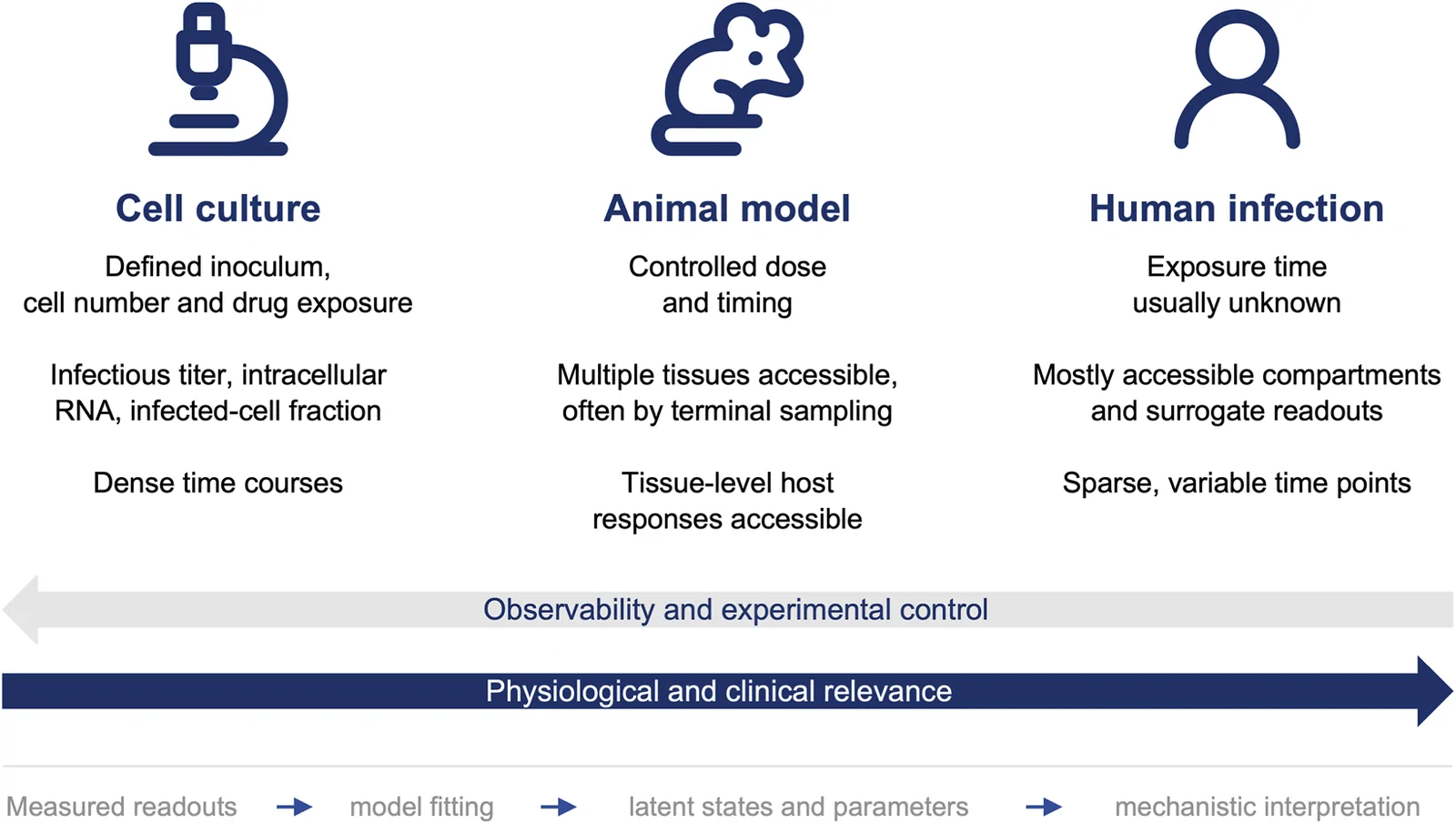
Experimental observability across biological scales. Cell-culture, animal and human infection systems differ in experimental control, accessible measurements and physiological relevance. As biological and clinical relevance increase, experimental observability generally decreases, constraining inference from measured readouts to latent biological states, parameters and mechanisms.

No individual assay provides a complete measurement of “the infection.” Instead, each experiment observes a particular component of a larger dynamic system, and different readouts may therefore respond differently to the same biological perturbation. Mechanistic viral-dynamics models provide a formal framework for connecting these experimentally accessible quantities to otherwise unobserved processes. The simplest such framework begins with the classical target-cell model of uninfected cells, infected cells and free virus.

## Mechanistic viral-dynamics models: from U-I-V to drug action

Mechanistic viral-dynamics models provide a framework for interpreting time-dependent measurements in terms of the biological processes that generate them. A large fraction of such models are formulated as systems of ordinary differential equations (ODEs), in which the state variables themselves change continuously with time. The classical target-cell-limited model considers three populations: susceptible or uninfected cells $U\left(t\right)$ , infected cells $I\left(t\right)$ , and free infectious virus $V\left(t\right)$ (De Boer and Perelson, 1998; Perelson, 1999). In its simplest form, their dynamics can be represented as

$$\frac{dU}{dt}=-\beta UV,$$

$$\frac{dI}{dt}=\beta UV-\delta I,\frac{dV}{dt}=pI-cV,$$

where β describes infection of susceptible cells, δ the loss of infected cells, p the production of infectious virus, and c its clearance. Numerous variations of this basic system exist, including models with target-cell proliferation or replenishment, but the U-I-V formulation remains a standard starting point for within-host and *in vitro* viral-dynamics modeling (Smith and Ribeiro, 2010; Perelson and Ribeiro, 2013; Zitzmann and Kaderali, 2018; Goyal et al., 2019). Solving the system also requires specification of its initial state. In controlled *in vitro* experiments, quantities such as initial cell number and inoculation conditions are often known, although the experimental inoculum need not correspond directly to $V\left(0\right)$ , particularly when adsorption is followed by washing and medium replacement. Such experimental steps can be incorporated when defining the effective starting state (Staroverov et al., 2023, 2024). In clinical infection, by contrast, the initial conditions are commonly unknown and may need to be estimated together with model parameters because neither the time nor the effective dose of infection is generally observed.

The central purpose of such a model is to translate an observed trajectory into quantitative hypotheses about the processes responsible for it. A viral growth curve by itself establishes that the measured quantity increased or decreased with time, but does not determine whether its shape was driven primarily by the rate of cell infection, the lifespan of infected cells, the production of new virus or viral clearance. Mechanistic modeling attempts to separate these contributions by assigning them explicit parameters and estimating values that are consistent with longitudinal observations. The classical HIV studies provide perhaps the most influential demonstration of this approach: analysis of viral-load decline following antiretroviral treatment revealed the rapid turnover of plasma virus and subsequently enabled estimation of virion clearance, productively infected-cell lifespan and viral generation time (Ho et al., 1995; Wei et al., 1995; Perelson et al., 1996, 1997). Similar reasoning was later used in HCV to separate the direct inhibition of viral production or release by interferon from the slower loss of infected cells (Neumann et al., 1998). Related treatment-perturbation approaches were applied to HBV following lamivudine administration (Nowak et al., 1996). At finer biological scales, multiscale influenza models have used longitudinal intracellular and extracellular measurements to assign observed infection dynamics to particular stages of viral replication and to predict the consequences of perturbing these stages pharmacologically (Heldt et al., 2013). This provides the first direct connection to antiviral drug development: rather than asking only whether a compound reduces a viral readout, a mechanistic model allows the more specific question of which process in the infection cycle is being perturbed, and by how much.

The standard U-I-V model is nevertheless a highly simplified description of viral infection (Perelson, 2002; Beauchemin and Handel, 2011; Smith and Perelson, 2011; Ciupe and Heffernan, 2017). It typically assumes homogeneous populations of susceptible and infected cells, immediate production of virus after infection, and constant rates of infection, production and clearance. It contains no explicit intracellular viral life cycle, spatial structure or immune response. In acute infections, viral decline is frequently explained primarily through depletion of susceptible cells, although *in vivo* clearance may instead involve substantial innate and adaptive immune control. These simplifications are not necessarily disadvantages. A minimal model provides a useful null hypothesis against which additional biological mechanisms can be evaluated and avoids introducing parameters that available data cannot support. However, its assumptions must be recognized when biological or pharmacological conclusions are drawn from fitted parameters.

Extensions of the basic model therefore generally correspond to specific biological hypotheses. One of the simplest additions is an eclipse compartment representing cells that have been infected but are not yet producing virus. In experimental influenza A infection in humans, inclusion of such a phase substantially improved the representation of the observed viral kinetics and led to an estimated delay of approximately 6 hours between infection and productive virus release (Baccam et al., 2006). The same study showed that a subset of trajectories with two viral-load peaks could be explained by extending the model with an interferon-mediated antiviral response (Baccam et al., 2006). More elaborate influenza models have incorporated innate and adaptive immune responses explicitly, including cytotoxic T cells and virus-specific antibodies, and have used corresponding experimental measurements to estimate their contributions to viral control (Lee et al., 2009; Miao et al., 2010).

At another level, intracellular models can describe viral genome replication, transcription, protein synthesis, assembly and release explicitly (Kumberger et al., 2016; Ciupe and Conway, 2024). Such models are especially attractive for antiviral research because individual drug targets often correspond much more naturally to intracellular processes than to the effective parameters of a simple target-cell model. A multiscale model of influenza A infection, for example, connected detailed intracellular viral replication with extracellular spread and was used to compare inhibition of viral entry, transcription, genome replication, protein synthesis, nuclear export and virion assembly or release (Heldt et al., 2013). The analysis predicted qualitatively different consequences of targeting different stages of the viral life cycle, including a primarily delayed infection following inhibition of entry compared with substantially reduced viral production when later intracellular processes were targeted (Heldt et al., 2013). Similarly, multiscale HCV models have connected intracellular replication to patient-level viral-load kinetics under direct-acting antiviral treatment and explored the effects of drugs acting at different viral targets (Clausznitzer et al., 2016). These examples demonstrate how model structure can be adapted to the pharmacological question being considered rather than treating all antiviral compounds as equivalent reductions in a single generic replication parameter.

Other extensions describe changes in target-cell susceptibility induced by innate immunity, direct immune-mediated killing of infected cells, antibody-dependent viral clearance, distinct susceptible and drug-resistant viral populations, or multiple anatomical compartments. Pharmacokinetic and pharmacodynamic components can additionally be introduced so that the antiviral effect varies with drug concentration over time rather than remaining constant. The resulting spectrum ranges from the minimal target-cell model to detailed multiscale descriptions of intracellular replication, immune responses and treatment (Canini and Perelson, 2014; Zitzmann and Kaderali, 2018). Importantly, increasing model complexity is not a goal in itself. Each additional state or parameter represents a biological hypothesis whose inclusion should ideally be justified both by the scientific question and by measurements capable of informing it.

Antiviral action can be represented by making the affected kinetic parameter concentration-dependent. For example, inhibition of viral production can be written as $p_{\text{eff}}\left(t\right)=\left[1-E\left(C\left(t\right)\right)\right]p$ , where $E\left(C\right)$ may be described by a Hill-type exposure-response function, $E\left(C\right)=E_{\max}C^{h}/\left({EC}_{50,PD}^{h}+C^{h}\right)$ . Analogous terms can be applied to infection, intracellular replication or other targeted processes. This formulation connects drug concentration and concentration-response shape to a defined perturbation of the viral-dynamics model and allows pharmacokinetics to be incorporated through $C\left(t\right)$ (Canini and Perelson, 2014).

This issue is particularly important when viral-dynamics models are used to quantify or predict antiviral efficacy. The same observed viral-load trajectory may be compatible with different mechanisms of infection control, and these mechanisms can generate different responses to treatment. Cao and McCaw compared influenza models in which viral decline was driven predominantly by target-cell depletion or by an innate immune response and showed that the assumed control mechanism substantially affected model-based estimates and predictions of antiviral treatment effects (Cao and McCaw, 2017). Thus, apparently satisfactory agreement with untreated viral-load data does not guarantee that a model will make reliable predictions after pharmacological perturbation. A model can reproduce an observed trajectory for the wrong biological reason.

This is also why dynamic modeling can provide information that is difficult to obtain from an endpoint EC50 alone. Once drug action is represented as inhibition of a particular process, the model can be used to evaluate how its consequences depend on treatment timing, dose and the stage of infection. Even relatively simple target-cell models can predict quantities such as peak viral load, time to peak, viral-load area under the curve and infection-associated target-cell loss, and can examine how these outcomes change when treatment is initiated at different times (Hadjichrysanthou et al., 2016). More detailed models can incorporate drug pharmacokinetics, resistance, immune responses or intracellular targets when these processes are necessary for the question being addressed.

However, mechanistic models cannot recover information that is absent from the data. Additional biological states introduce additional parameters, but different parameter combinations or mechanisms may generate nearly indistinguishable observations. The mechanistic value of a model is therefore constrained not only by biological plausibility but also by experimental observability. Determining which parameters can actually be inferred from a given set of measurements is the subject of the following section.

## From measurements to parameters: the inference problem

The biological model and the measurement process represent two distinct components of parameter inference. A dynamical model generates trajectories of latent state variables such as U, I, or V, whereas an experiment produces observations that are related to these variables through a particular assay. This relationship can be described by an observation model. In general, if $x\left(t\right)$ denotes the vector of biological state variables, an experimental measurement $y_{k}$ obtained using assay k can be represented schematically as

$$y_{k}\left(t\right)=h_{k}\left(x\left(t\right),\theta ,s_{k}\right)+\varepsilon_{k}\left(t\right),$$

where $h_{k}$ describes how the biological states are converted into the measured quantity, θ denotes the vector of model parameters, $s_{k}$ represents assay-specific scaling or calibration parameters, and $\varepsilon_{k}\left(t\right)$ represents measurement error. Thus, parameter estimation requires fitting not only a model of infection dynamics but, explicitly or implicitly, a model connecting those dynamics to the experiment. This distinction is important because a good correspondence between a model trajectory and experimental data does not necessarily imply that the measured quantity is identical to the corresponding biological state.

For the classical U-I-V model, infectious-virus assays provide the closest experimental proxy for V, although this correspondence remains assay-dependent. PFU, focus-forming units and TCID50 quantify infectious activity under defined experimental conditions rather than the absolute number of physical virions (Cresta et al., 2021). A model can therefore define V directly in assay units, but parameters involving V, including infection and production rates, then inherit this scale. Other readouts map still less directly onto the classical state variables: extracellular and intracellular RT-qPCR quantify different pools of viral material, flow cytometry or immunostaining may approximate the fraction rather than the absolute number of infected cells, and viability measurements generally reflect the total viable population rather than U alone. Immune measurements require corresponding biological states if they are to be interpreted mechanistically (Miao et al., 2010). Thus, the observation model should reflect what the assay actually reports rather than assuming that an experimental readout is identical to a latent model variable.

Scaling between a biological state and its experimental measurement creates an additional, sometimes underappreciated, difficulty (Holder et al., 2011). A mathematical model may conceptually describe virions or infected cells, whereas experimental data may be reported as TCID50/ml, PFU/ml, RNA copies/ml, fluorescence units or percentages of positive cells. The conversion between these units is frequently unknown. If an experimental viral measurement is related to the underlying viral population by a scaling factor, this scaling can directly affect the apparent values of other model parameters. For the simple target-cell model, rescaling V changes the corresponding numerical values of both the infection parameter β and virus production parameter p , even though the predicted trajectory in experimental units can remain unchanged. Consequently, a parameter reported as a virus production rate may partly reflect assay calibration rather than an intrinsic biological quantity.

This problem has been examined explicitly in influenza modeling. Petrie et al. introduced scaling relationships between biological viral populations and measurements obtained by TCID50 and RT-qPCR and showed that assay sensitivity and calibration could systematically affect estimated kinetic parameters (Petrie et al., 2013). Unknown scaling factors can therefore become additional parameters in the inference problem and may be correlated with biologically interesting parameters such as burst size, infection rate or viral production. Where possible, independent calibration of these relationships is preferable to estimating all scaling factors jointly with the dynamic parameters.

Once biological and observation models have been specified, unknown parameters are estimated by comparing model predictions with longitudinal measurements. Approaches used in viral-dynamics studies include nonlinear least-squares, maximum-likelihood and Bayesian methods, while nonlinear mixed-effects models are particularly useful for separating population-level parameters from inter-individual variability. These approaches have recently been reviewed in detail (Tower et al., 2026). Importantly, the statistical observation model must also account for properties of the data, including multiplicative variation across orders of magnitude and measurements below or above assay quantification limits, because assumptions concerning error and censoring can affect parameter estimates.

Irrespective of the fitting algorithm, however, successful optimization does not imply that the underlying parameters have been uniquely determined. This distinction is captured by the concept of parameter identifiability. Structural identifiability asks whether a parameter can, in principle, be uniquely recovered from ideal, continuous and noise-free observations of the specified model outputs. If multiple parameter sets produce exactly the same observable trajectory, the model is structurally unidentifiable with respect to those parameters, and no improvement in optimization or additional measurements of the same output can uniquely recover them. Practical identifiability asks whether structurally identifiable parameters can actually be estimated with useful precision from the finite, noisy and incompletely sampled data available in a real experiment (Cobelli and DiStefano, 1980; Raue et al., 2009; Miao et al., 2011; Simpson and Maclaren, 2023; Murphy et al., 2024; Tuncer et al., 2025).

Identifiability is a particularly important issue for viral-dynamics models because the parameters of even simple ODE systems can be strongly correlated. For example, changes in the infection rate, initial target-cell abundance and virus production rate can compensate for one another and generate very similar viral-load trajectories. As biological mechanisms are added, the number of such compensating relationships can increase. Consequently, a more detailed model may provide a better numerical fit while producing less reliable estimates of individual biological processes. Structural and practical identifiability analyses can reveal these limitations through analytical methods, profile likelihoods, sensitivity analyses, posterior distributions or examination of parameter correlations (Miao et al., 2011; Tuncer et al., 2025). Mechanistic complexity should therefore be limited not only by biological plausibility, but also by experimental observability.

One of the most effective ways to improve observability is to measure distinct components of the infection process simultaneously. If only infectious V is observed, changes in cell infection, intracellular replication, particle production and loss of infected cells can potentially generate similar viral trajectories. Measurement of infected cells provides an independent constraint on the rate at which infection spreads through the cell population. Simultaneous quantification of total viral genomes and infectious virus can help distinguish production of viral material from production of replication-competent progeny. Intracellular viral RNA provides additional information about replication before release, while direct cell counts or viability measurements constrain changes in the host-cell population. Immune measurements can similarly constrain models in which viral decline is attributed to specific host responses rather than target-cell depletion.

Iwami et al. provided an early demonstration of this principle by simultaneously measuring infectious viral load, total viral RNA, and the fractions of infected and uninfected cells during *in vitro* SHIV infection at four different multiplicities of infection (Iwami et al., 2012). Joint fitting of these measurements allowed estimation of the infected-cell half-life, infectious-virus half-life, total and infectious burst sizes, and the ratio between infectious and total virions. Importantly, the estimated production of total viral particles was far greater than that of infectious virus, a distinction that could not have been recovered by treating a single measurement as generic viral load. In an influenza ferret model, simultaneous fitting of infectious-virus titers and total viral RNA likewise reduced uncertainty and improved consistency of several parameter estimates compared with fitting infectious-virus measurements alone (Petrie et al., 2013). Multimodal SARS-CoV-2 experiments have similarly shown that infectious titers, intracellular RNA, infected-cell percentages and viability measurements can jointly reject otherwise plausible model structures that fit only a subset of the observations (Staroverov et al., 2023).

However, the number of measured variables is not itself a useful measure of experimental informativeness. Several highly correlated assays may constrain essentially the same combination of parameters, whereas a single additional measurement of a previously unobserved state can fundamentally change what can be inferred (Holder and Beauchemin, 2011). The relevant question is therefore not how many measurements can be collected, but which measurements distinguish the competing mechanisms of interest. This principle is especially important for antiviral drug development. If the objective is to determine whether a compound inhibits genome replication or reduces the infectivity of released particles, for example, adding another assay proportional to extracellular viral RNA may be less informative than measuring intracellular RNA together with infectious-virus output.

Parameter inference and experimental design should consequently be considered an iterative process. An initial model can be fitted to available data and analyzed to identify parameters or mechanisms that remain poorly constrained. These results can then motivate additional measurements or experimental perturbations designed specifically to separate competing explanations. Such perturbations can include different multiplicities of infection, treatment concentrations, times of drug addition, washout experiments or comparison across biological systems. The new measurements can subsequently be used to refine or reject the original model. Formal Bayesian experimental-design approaches have been developed for viral-dynamics studies (Huang and Wu, 2008), while identifiability analyses provide a general framework for selecting measurements and sampling schemes that improve parameter estimation (Miao et al., 2011; Tuncer et al., 2025). Model-driven experimental design has also become an important component of experimental influenza research, where predictions generated by fitted models have been tested through subsequent laboratory perturbations (Smith, 2018).

The effectiveness of this iterative approach depends on experimental access to the relevant biological states. Controlled *in vitro* systems permit dense multimodal measurements and targeted perturbations, although complete parameter identification can remain difficult even under these conditions. In naturally infected humans, many cellular, intracellular and immune states are inaccessible, while viral measurements are typically sparse and compartment-specific. The inference problem therefore becomes substantially more difficult as experimental control and observability decrease.

## Why human viral dynamics are hard to identify

Human infection represents the setting of greatest clinical interest for antiviral development, but also one of the least experimentally observable. In naturally acquired infections, the time of infection and effective inoculum are usually unknown, and sampling often begins only after symptoms develop or a diagnostic test becomes positive. For acute respiratory infections, this may occur near or after the peak of viral replication. Measurements are then collected at relatively sparse and irregular intervals, frequently from a single anatomical site, requiring the early phase of infection to be reconstructed from incomplete observations.

SARS-CoV-2 provided an unusually large amount of longitudinal human viral-load data, but also clearly demonstrated these limitations. A pooled analysis of 2,172 upper-respiratory viral-load measurements from 605 individuals showed variation exceeding five orders of magnitude among individuals sampled at similar times after symptom onset (Challenger et al., 2022). Intensively sampled prospective cohorts provide considerably more information: daily longitudinal sampling, for example, has enabled estimation of individual viral expansion and clearance rates and demonstrated substantial person-to-person variation in the magnitude and duration of infectious-virus shedding (Ke et al., 2022). Nevertheless, such datasets remain exceptional compared with routine clinical sampling, and even dense measurements generally describe only one or several accessible compartments rather than the complete infection.

Anatomical sampling can also reveal biological compartmentalization within the host. Sequencing of paired longitudinal saliva and nasal samples demonstrated significant within-host genetic compartmentalization between these sites in multiple individuals (Farjo et al., 2024). Thus, a measured viral-load trajectory may reflect local replication and shedding within a spatially heterogeneous infection rather than a single host-wide viral population.

An even greater source of uncertainty is that most of the host processes responsible for controlling infection are not observed directly. Viral decline in an infected human can potentially reflect depletion or reduced susceptibility of target cells, interferon-mediated induction of an antiviral state, innate immune effector mechanisms, antibody-mediated neutralization, killing of infected cells by adaptive immune responses, or several of these processes acting concurrently. However, many viral-dynamics datasets contain only serial viral RNA measurements. Incorporating an immune-response compartment into a model does not by itself provide information about that response if the corresponding biological measurements are unavailable.

The consequences of this problem can be demonstrated formally. Sachak-Patwa et al. considered an influenza model in which an explicit immune response generated the observed viral dynamics and showed that its parameters could be estimated when viral load, cell-death and immune-response measurements were available. When only viral load was retained, the immune-response model became poorly identifiable, whereas substantially simpler target-cell-limited models could reproduce the same characteristic rise and decline of virus (Sachak-Patwa et al., 2023). Moreover, parameters of the simple models changed systematically as the unobserved immune response in the data-generating model changed. Thus, a target-cell-limited model can provide an accurate phenomenological description of viral kinetics even when target-cell depletion is not the biological mechanism responsible for viral control. This distinction is important whenever fitted parameter differences are interpreted as mechanistic differences between patients, viral variants or treatment groups.

The same issue is evident in the SARS-CoV-2 modeling literature. Models fitted to upper-respiratory viral-load data have incorporated type I interferon responses, nonspecific innate immunity, humoral immunity and cell-mediated responses, but estimates of key viral-dynamic parameters differ substantially among studies (Du et al., 2022). This uncertainty does not imply that models containing immune processes are uninformative. Rather, conclusions concerning a particular immune mechanism should reflect the extent to which that mechanism is independently constrained by data. In the absence of such measurements, the same model component should be interpreted more cautiously as an effective representation of host-mediated viral control.

Inter-individual heterogeneity further complicates inference from human viral-load trajectories. Patients differ in age, previous infection, vaccination history, baseline immune state, infecting variant, timing of treatment and numerous other host characteristics. These factors can alter the observed viral phenotype independently of intrinsic viral replication kinetics or drug susceptibility. This became particularly important as population immunity against SARS-CoV-2 accumulated. In a prospective cohort containing infections with several variants, a hierarchical model jointly accounting for viral variant, age and prior antigen exposures showed that previous vaccination and infection history substantially influenced estimated shedding dynamics (Russell et al., 2024). Consequently, differences observed between Delta and Omicron infections could not be interpreted without accounting for the changing immune backgrounds of the individuals in whom those variants occurred. For antiviral studies, the analogous problem is that variation in treatment response can reflect both pharmacological activity and heterogeneity of the infected hosts.

Despite these limitations, sparse human viral-load measurements can still support clinically useful inference. The distinction is primarily between what can be estimated or predicted from the observed trajectory and the strength of the biological interpretation attached to those estimates. Viral-dynamics models have, for example, been used to reconstruct quantities that are otherwise difficult to observe directly. Ejima et al. used longitudinal SARS-CoV-2 viral loads to hindcast the timing of infection and thereby estimate the incubation-period distribution (Ejima et al., 2021). Related simulations based on fitted viral-load dynamics have been used to compare strategies for ending isolation (Jeong et al., 2021) and to investigate the design of antiviral clinical trials (Iwanami et al., 2021). In the latter study, substantial heterogeneity in untreated viral kinetics reduced the statistical power to detect antiviral effects, whereas restricting enrollment to patients treated shortly after symptom onset markedly reduced the predicted sample size required. These applications depend primarily on reproducing clinically relevant features of the viral-load trajectory rather than on uniquely identifying every biological process that generates it.

The same principle applies when viral-dynamics models are used to investigate treatment. Comparison of longitudinal viral-load data for SARS-CoV-2, SARS-CoV and MERS-CoV was used to estimate differences in their within-host dynamics and to simulate hypothetical antiviral mechanisms, predicting a strong dependence of treatment benefit on the timing of administration relative to the viral-load peak (Kim et al., 2021a). Related modeling of SARS-CoV-2 infection in non-human primates predicted that incomplete inhibition initiated early in infection could, under some conditions, prolong the duration of viral shedding despite reducing viral replication (Kim et al., 2021b). Human models have likewise been used for more direct pharmacological applications. Zhang et al. compared eight SARS-CoV-2 viral-dynamics models using 2,155 measurements from 162 individuals and subsequently used the selected model to simulate treatment with tixagevimab-cilgavimab (Zhang et al., 2023). Quantitative systems pharmacology models fitted to human viral kinetics have also been used to compare antiviral interventions and support clinical dose selection (Claas et al., 2024).

These examples illustrate that predictive or decision-making utility does not require every internal model parameter to have a unique mechanistic interpretation. Conversely, successful reproduction of viral-load trajectories or clinically relevant outcomes does not by itself validate the biological mechanism assumed by the model. For example, fitting models with different transmission processes to viral-load trajectories from mild and severe COVID-19 cases suggested an association between cell-to-cell viral transmission and disease severity (Odaka and Inoue, 2021). Such results can generate useful mechanistic hypotheses, but when the relevant cellular processes are not measured independently, their interpretation remains conditional on the assumed model structure. Viral-load models can therefore provide robust information about quantities such as infection timing, viral growth and decline, treatment windows or expected trial outcomes while offering substantially weaker evidence for the specific intracellular or immune mechanisms responsible for those dynamics (Crespi et al., 2007; Clapham et al., 2014).

Human challenge studies occupy an unusual position between observational human studies and controlled experimental systems. Because exposure occurs at a defined time with a known inoculum, sampling can begin before detectable viral replication and continue at high temporal resolution. In the first SARS-CoV-2 human challenge study, healthy young adults were inoculated intranasally with a defined viral dose and followed using both viral RNA and infectious-virus measurements (Killingley et al., 2022). Modeling of these data estimated doubling times of approximately 2 h for viral RNA and 3 h for infectious virus and compared models incorporating target-cell limitation, innate immunity and adaptive immunity (Iyaniwura et al., 2024). The same challenge framework also enabled single-cell and multi-omics characterization of local and systemic immune responses (Lindeboom et al., 2024), providing unusually rich data for constraining mechanistic models. However, challenge studies are necessarily restricted to pathogens and participant populations in which deliberate infection can be performed safely and ethically and may not capture the heterogeneity of naturally acquired infection.

Human studies therefore present a fundamental trade-off. They provide the measurements most directly relevant to clinical efficacy while offering the least experimental control over initial conditions and only partial access to the biological states that generate the observed data. Their greatest strength may consequently lie in estimating population-level and clinically relevant properties of infection and treatment, while detailed mechanistic hypotheses often require complementary evidence from more experimentally accessible systems. Human studies maximize clinical relevance while minimizing experimental control and observability; as model complexity increases, it can therefore rapidly exceed the mechanistic information contained in the available data. Controlled *in vitro* infection systems occupy the opposite end of this spectrum and provide an opportunity to examine how much more can be learned when both the infection and its experimental observation can be manipulated directly.

## *In vitro* infection models: controlled systems, complex biology

The limitations of human data make *in vitro* infection systems particularly attractive for mechanistic viral-dynamics modeling. The time and conditions of inoculation can be controlled, viral and cellular quantities can be sampled densely, and multiple measurements can be obtained from parallel cultures under nearly identical conditions. Experimental variables such as multiplicity of infection (MOI), cell density, drug concentration and treatment timing can also be deliberately perturbed. *In vitro* experiments therefore provide an opportunity not only to quantify whether an antiviral compound reduces infection, but also to determine which biological processes are altered by treatment.

However, increased experimental control does not imply that viral dynamics become biologically simple. Parameters such as cell-entry rate, duration of intracellular replication and production of infectious progeny are properties of a particular virus-cell system rather than intrinsic constants of the virus. SARS-CoV-2 provides a clear example. Replication kinetics and infectious-virus production differ substantially among commonly used Vero E6, Caco-2 and Calu-3 cultures, as well as among viral variants within the same cellular system (Mautner et al., 2022). Similar strain-specific differences have been resolved mechanistically for influenza A virus: modeling infection of differentiated human bronchial epithelial cultures attributed differences among pandemic H1N1, seasonal H1N1 and avian H5N1 viruses to differences in quantities including viral production rate and the within-culture basic reproduction number (Mitchell et al., 2011). Later work extended this approach across multiple influenza A strains, showing how differences in observed *in vitro* growth rates could be decomposed into mechanistic traits such as the within-culture reproduction number and mean generation time (Yan et al., 2020). These cell lines differ in the availability of entry pathways and innate antiviral responses. In particular, Vero cells carry a homozygous deletion encompassing the type I interferon gene cluster and consequently cannot produce type I interferon (Osada et al., 2014); Vero E6 cells nevertheless remain responsive to exogenous IFN-I, which can substantially attenuate SARS-CoV-2 replication (Lokugamage et al., 2020). Such differences can alter both viral kinetics and the apparent effect of pharmacological perturbations.

The complexity of even a controlled cell-culture infection was illustrated by modeling SARS-CoV-2 infection in Caco-2 cells using infectious-virus titers, intracellular viral RNA, cell viability and infected-cell measurements (Staroverov et al., 2023). Standard target-cell-limited models could not explain several qualitative features of these data, particularly the decline in viral production in the absence of substantial cytopathic loss of the cell population. A model incorporating exhaustion of intracellular resources required for viral production and cytokine-mediated conversion of susceptible cells into a resistant state provided a substantially better description of the observations (Staroverov et al., 2023). Importantly, the need for these additional mechanisms was supported by several independent experimental observables rather than by improved fitting of a single viral-load curve. In this setting, failure of a simple model was itself informative because multimodal measurements restricted the range of biologically plausible explanations.

Comparison across cellular systems can provide additional mechanistic information. In a subsequent analysis, the dynamics of Delta and Omicron SARS-CoV-2 were modeled in both Caco-2 and Calu-3 cells using infectious-virus titers and intracellular viral RNA (Staroverov et al., 2024). A model in which variants differed only in cell-entry rate was insufficient to explain the observations. Instead, accurate description of both variants and both cell lines required differences in parameters associated with entry and cytokine-mediated antiviral activity, with Omicron characterized by a lower estimated entry rate and stronger induction of the modeled antiviral response (Staroverov et al., 2024). Thus, differences among cell systems need not be considered solely as unwanted experimental variability. When the relevant cellular biology is understood, comparison of distinct cell types can function as a controlled perturbation of host processes and help distinguish mechanisms that would remain confounded in a single system.

Influenza modeling provides complementary examples of how rich *in vitro* measurements can support models at finer biological scales. Heldt et al. integrated intracellular viral RNA and protein measurements with cell-population dynamics, allowing intracellular replication and extracellular spread to be represented within the same multiscale framework (Heldt et al., 2013). Related work subsequently showed that such models could describe influenza replication across markedly different MOI regimes while accounting for differences between total and infectious virus production (Rüdiger et al., 2019). These studies illustrate how controlled *in vitro* systems can separate processes that are difficult to observe independently *in vivo*.

Pharmacological perturbation can provide an additional source of information (Beauchemin et al., 2008). Rather than fitting untreated viral growth alone, experiments can compare several drug concentrations or treatment times, forcing competing mechanistic hypotheses to generate different predictions. A recent SARS-CoV-2 study combined time-resolved infections of A549-ACE2 cells with endpoint concentration-response measurements of the sigma-receptor ligand PB28 (Lisowski et al., 2026). The model jointly estimated viral kinetic and pharmacodynamic parameters and attributed the drug effect to reduced viral production. Notably, the apparent IC50 inferred from the time-resolved experiments was approximately twofold higher than that obtained from conventional endpoint assays (Lisowski et al., 2026), directly illustrating the dynamic nature of antiviral potency discussed earlier.

The mechanistic advantages of *in vitro* systems must nevertheless be balanced against their biological limitations. Immortalized or engineered cell lines can differ substantially from the tissues they are intended to represent in receptor abundance, protease usage, metabolism and immune signaling. The experimental system can even modify the virus itself. During passage in Vero E6 cells, SARS-CoV-2 rapidly selects spike variants with altered S1/S2 cleavage properties that improve utilization of available cellular proteases but reduce entry efficiency in TMPRSS2-positive human cells (Chaudhry et al., 2022). Thus, the apparently well-controlled experimental system can change the biological phenotype being measured.

*In vitro* systems therefore maximize experimental control and observability, but the mechanisms and parameter values inferred from them remain conditional on the cellular environment in which they were obtained. For antiviral development, the relevant question is consequently not which cell line represents human infection in an absolute sense, but whether the experimental system contains the biological process targeted by the compound and whether the collected measurements can distinguish its perturbation from competing mechanisms. When these requirements are met, dynamic *in vitro* experiments provide a powerful intermediate step between conventional antiviral screening and the substantially less observable conditions encountered in animals and patients.

## From viral dynamics to antiviral drug development

The ultimate value of viral-dynamics modeling for antiviral development is its ability to connect pharmacological exposure with a perturbation of the viral life cycle and, subsequently, with the observed course of infection (Ciupe, 2018; Hill et al., 2018). Historically, hepatitis C virus (HCV) provides one of the clearest examples of this approach. Viral-kinetic models were used throughout the development of interferon-based and direct-acting antiviral therapies to quantify treatment effectiveness, investigate mechanisms of action and predict treatment outcomes (Guedj et al., 2010; Rong and Perelson, 2010; Chatterjee et al., 2012, 2013; Canini and Perelson, 2014; Nguyen and Guedj, 2015). In one particularly direct application, a model integrating *in vitro* virology, pharmacokinetics, viral resistance and early clinical viral kinetics during treatment with the protease inhibitor telaprevir was parameterized using early-phase studies and subsequently prospectively predicted sustained virological response rates observed in later trials with different treatment durations and patient populations (Adiwijaya et al., 2012). More detailed HCV models have additionally separated effects on intracellular RNA replication, degradation and viral secretion. For example, analysis of patients receiving the protease inhibitor danoprevir estimated strong inhibition of intracellular viral production together with increased RNA degradation and partial inhibition of viral secretion (Rong et al., 2013). These examples demonstrate that treatment-induced dynamics can provide information extending beyond an endpoint reduction in viral load, although mechanistic interpretations are strongest when supported by independent knowledge of the molecular drug target.

Integration with pharmacokinetics provides the next step toward clinical decision-making. Instead of describing antiviral efficacy by a constant parameter, drug inhibition can be linked to a time-dependent concentration C(t), allowing the consequences of dose, dosing interval and treatment duration to be simulated. An unusually clear recent example is the development of nirmatrelvir/ritonavir. Phase I pharmacokinetic data, nonclinical antiviral measurements and a quantitative systems pharmacology model were jointly used to support selection of 300/100 mg twice daily for phase II/III evaluation; the model also predicted that a 5-day regimen would substantially reduce SARS-CoV-2 viral load (Singh et al., 2022). Thus, in this case modeling was not merely a retrospective explanation of an approved treatment but formed part of the evidence used to select the regimen taken into pivotal clinical trials.

Influenza provides another example in which viral dynamics have been combined with clinical pharmacology. A population drug-disease model based on data from 208 subjects receiving placebo or different doses of oseltamivir represented the drug as inhibition of viral production and reproduced the limited additional virological benefit predicted from doubling the standard dose (Kamal et al., 2015). Subsequent analysis in immunocompromised adults integrated oseltamivir pharmacokinetics, exposure-response relationships and influenza viral dynamics. No substantial additional antiviral benefit was identified at exposures above those produced by the conventional 75-mg dose, whereas model simulations supported early initiation and a longer, 10-day course to reduce the risk of viral rebound (Sturm et al., 2021). These results supported use of 75 mg twice daily for 10 days in this population.

Models can also identify questions that remain unresolved after a treatment has entered clinical use. For nirmatrelvir, a viral-immune model coupled with pharmacokinetics was later used to reproduce clinical viral-load trajectories and investigate post-treatment rebound (Esmaeili et al., 2024). The analysis suggested that effective *in vivo* potency was lower than predicted from *in vitro* assays and that extending treatment duration could reduce rebound more effectively than increasing dose or dosing frequency. These remain model-based predictions rather than established changes to clinical practice, but they illustrate how the same framework can generate testable hypotheses concerning treatment timing and duration.

The available examples therefore suggest a more focused role for viral-dynamics models in antiviral development than replacing conventional potency assays or clinical trials. Their greatest utility arises when sufficiently informative longitudinal data allow drug exposure to be linked to a defined process in the infection model. Under these conditions, modeling can help characterize mechanism and magnitude of antiviral action, support dose and regimen selection, and explain why treatment effects observed *in vitro* do not translate directly to patients. The main value of viral-dynamics modeling in drug development is therefore not prediction alone, but the ability to connect drug exposure to a defined biological perturbation and then to the resulting infection trajectory.

## Discussion

Antiviral effects are observed through experimental measurements that capture only particular components of an evolving infection. Infectious-virus titers, viral RNA, infected-cell measurements, viability assays and immune readouts therefore cannot be interpreted as interchangeable representations of a single quantity. Likewise, endpoint measures such as EC50 and EC90 are shaped not only by molecular drug activity but also by viral replication kinetics, host-cell biology, assay design and the timing of measurement. Interpretation of antiviral activity consequently requires consideration of both the biological system and the experimental process through which it is observed.

Mechanistic viral-dynamics models provide a framework for connecting these observations to processes such as cell infection, intracellular replication, production of infectious progeny, cell loss and host-mediated control. However, the mechanistic information obtained from such models is ultimately limited by observability and identifiability. Increasing biological detail cannot compensate for measurements that do not distinguish competing mechanisms. Conversely, appropriately selected longitudinal and multimodal measurements can allow relatively simple models to discriminate among biologically meaningful explanations of infection dynamics.

An additional limitation is that model-derived predictions are not necessarily experimentally or clinically validated. Agreement with the data used for model fitting does not constitute prospective validation, and predictions concerning unobserved mechanisms, treatment responses or optimal intervention strategies remain conditional on the assumed model structure and parameterization. Experimental perturbations, independent datasets and, where appropriate, prospective clinical evaluation are therefore needed to distinguish robust predictions from plausible but model-dependent hypotheses. Mechanistic models are consequently most useful when they generate testable predictions that can be iteratively challenged and refined by experiment or clinical observation.

This relationship between biological relevance and experimental observability changes across experimental scales. Controlled *in vitro* systems permit dense measurements and targeted perturbations, whereas animal and particularly human studies provide greater physiological relevance with progressively more limited access to the underlying infection states. For antiviral development, the most informative strategy is therefore not to maximize either experimental or mathematical complexity in isolation, but to align the biological system, measurements and model with the pharmacological question being addressed. Such integration allows antiviral effects to be interpreted as dynamic consequences of defined biological perturbations rather than as properties of individual experimental readouts alone.
